# Recent advances in treatment for Benign Prostatic Hyperplasia

**DOI:** 10.12688/f1000research.7063.1

**Published:** 2015-12-21

**Authors:** Simon van Rij, Peter Gilling

**Affiliations:** 1 Guy’s & St. Thomas NHS Trust, Great Maze Pond, London, SE1 9RT, UK; 2 Tauranga Hospital, Tauranga, New Zealand

**Keywords:** benign prostatic hyperplasia, personalised medicine, prostate obstruction, aquablation, mirabegron, Holmium laser enucleation of the prostate, HOLEP

## Abstract

Clinical benign prostatic hyperplasia (BPH), often identified as a worsening ability of a male to pass urine, is a significant problem for men in our society. In 2015, the use of personalised medicine is tailoring treatment to individual patient needs and to genetic characteristics. Technological advances in surgical treatment are changing the way BPH is treated and are resulting in less morbidity. The future of BPH treatments is exciting, and a number of novel techniques are currently under clinical trial.

Clinical benign prostatic hyperplasia (BPH), often identified as the worsening ability of a male to pass urine, is a multi-billion-dollar industry ^1^. BPH increases with age and therefore with an aging society the incidence will continue to increase significantly. BPH is both extremely common and can cause significant harm ^2^. Treatment options for men with prostate obstruction have existed for centuries. But what is new in 2015? What is making this branch of medicine such an exciting area to be working and researching in? Much of it is to do with the personalised medicine mantra and the medical technology revolution. Using these two key principles, we will show the changing nature of the treatment of BPH.

BPH is often used as a colloquial term to describe urinating difficulties and bother in men. However, within the literature, a number of different terms and acronyms are used to describe the symptoms and conditions associated with urinating difficulties ^3^. Strictly speaking, BPH is a pathological change that occurs in the prostate, leading to enlargement. This enlargement can lead to obstruction now termed benign prostatic obstruction (BPO). BPO can lead to urinary symptoms; however, one must remember that a number of other conditions can cause urinary symptoms in men. Both the European Association of Urology and the American Urology Association publish clear evidence-based guidelines on these subjects ^4, 5^. To keep this review focused, we will stick to advances in BPH and BPO.

‘Personalised medicine’ has become a buzzword in the management of many medical conditions ^6^. It involves moving away from a one-size-fits-all treatment. Instead, it is a process of weighing up the benefits of different treatments for an individual on the basis of their own specific characteristics. Much of this has grown from our increased understanding of genetics and the human genome. To apply this to BPH, we must look at recent advances in the pathogenesis of this condition.

The effect of androgens on the development of BPH has been well studied ^7^. However, as our understanding of the topic has increased, so too has the complexity of this condition. Inflammation, growth factors, stromal interactions, and genetic factors have all been shown to contribute to the hyperplasia of the prostate glands and stroma. Much of the current research has focused on the gene expression that differs between individuals, particularly around cellular pathways and receptors. The enzyme 5-alpha reductase 2 (5AR2) plays a key role in the conversion of androgens in the prostate, leading to hyperplasia ^6^. Epigenetic studies have shown that the expressions of these 5AR2 proteins are varied amongst subjects and appear to be linked to the development of BPH ^8^. The exciting prospect for the future is developing commercial tests to identify these proteins in individuals to tailor medication to that individual.

Management of BPH has often been divided into medical and surgical options. However, in 2015, the lines between the two are now more blurred. This has been driven by patients’ expectations of their treatments and new technology making treatments less invasive. In the last year, no new medications that specifically act on the prostate have been brought to commercial market. Alpha-blockers, which cause relaxation of the smooth muscle fibers within the prostate, continue to be the first-line treatment ^5^, although the drugs have no impact on the progression of BPH or on the potential to avoid surgical treatment ^9^. Also, recent research shows that only 40% of patients commenced on these medications remain on treatment 6 months later ^10^. An alternative medication group are the 5-alpha reductase inhibitors. Large multi-centre randomised controlled trials have shown the benefit of these medications in improving urinary symptoms ^9^. However, evidence in relation to the side effects of these drugs has surfaced over the last few years. The most publicised came from the Prostate Cancer Prevention Trial, in which men on finasteride showed a possible increase in high-risk prostate cancer compared with those on placebo ^11^. The absolute difference in cancer rates was extremely low, and subsequent commentaries and analysis have aimed to disprove this ^12^. The other concerning side effect of finasteride has been a reported worsening sexual function, which in some men can be longstanding ^13^. Finasteride given at a lower dose is a common medication used for male hair loss, and reports have shown these same concerns on sexual function when used for this purpose ^14^. The only new medications on the market for BPH in the last few years have been the phosphodiesterase type 5 (PDE5) inhibitors, most notably tadalafil. The most common indication for this medication is the treatment of erectile dysfunction, but trials have shown improvement in BPH symptoms without adverse sexual side effects ^15^. The exact mechanism for the effect of tadalafil for symptomatic BPH still has not been elucidated ^16^. It is possible that it has little effect on BPH but that because men have improved sexual function they feel better!

Apart from traditional oral medication, attempts have been made to inject drugs directly into the prostate. This has been spurred on by the increased use and efficacy of Botox (Allergan, Dublin, Ireland) treatment in the bladder ^17^. Initial trials in intraprostatic injection have been promising. However, the only long-term randomised controlled trial of intraprostatic Botox did not show significant benefit ^18^. Other novel agents are being explored but still require further evidence before they can be used clinically. As definitive trials have not yet been published, it is possible that they have not resulted in adequate clinical effectiveness ^19^.

Technology has played a significant role in the latest advances in BPH management, no more so than in the increase in interventions that do not require general anaesthetic or lengthy stays in hospital. A number techniques, known as ‘office procedures’, have involved different forms of energy, including heat and water. The most promising new technique has been the prostatic urethral lift. This is a novel mechanical implant placed into the prostate that pulls the encroaching lobes of the prostate out of the way to improve men’s flow ^20^. As discussed previously, the effect of treatment on sexual function can be a key determinant for a patient deciding what treatment option to have. From a surgical point of view, most procedures result in retrograde ejaculation, which can affect both fertility and sexual performance/satisfaction. The UroLift device has been shown to cause minimal disruption to ejaculation and, when compared with the traditional standard form of surgery—transurethral resection of the prostate (TURP)—in a recent trial, had similar outcomes, including patient satisfaction with minimal complications ^21^. Many of these novel techniques are introduced early without long-term data to show that they remain efficacious. It remains to be seen whether the UroLift will became a mainstay of treatment or fall by the wayside like other techniques before it.

A more controversial new technique with only relatively recent published data is prostatic artery embolisation. Performed by an interventional radiologist rather than a urologist, this technique is performed under local anaesthetic and involves a groin artery puncture with super-selective vascular embolisation of the arteries to the prostate. This is postulated to cause shrinkage of the prostate and an improvement in urinary function. To date, this technique has been hampered by poor study design with no comparative randomised controlled trials published ^22^. An article published this year from China shows that, in men with enlarged prostates, a significant decrease in the size of prostates along with symptom improvement is obtained ^23^. The jury remains out on this technique, and professional societies have published guidelines cautioning mainstream use until proper rigorous data are published ^24^. A number of ongoing trials will be reported in the next two years, and so we will wait and see.

TURP has long been the standard of care for the surgery of BPH unless the prostate was very large and in that case an open operation was performed. The goal with technology has been to improve upon these techniques to provide better outcomes with less morbidity. In 2015, the surgical options open to a patient are many, including traditional surgery, laser surgery, and in some cases robotic surgery. The key questions are whether the technology is actually better and the role of device companies as a driving force.

Lasers have been used in endoscopic BPH surgery for over 20 years ^25^. The unique properties of each laser and its individual wavelength allow precise cutting and vaporisation of tissue with excellent haemostasis. Techniques to remove prostate BPH tissue fall broadly into ablative techniques with destruction of the tissue versus enucleation techniques, whereby tissue is shelled out in large anatomic lobes for removal. Although these techniques have been around for a long time, only recently have they become the standard of care when compared with traditional surgery ^26^. This has come about from well-designed trials comparing laser techniques with traditional surgery. The GOLIATH trial compared the 180-W version of the 532-nm laser (often called the ‘greenlight’ laser) with traditional surgery within a randomised control trial. The results at two years showed non-inferiority of the laser with lower rates of complications, particularly in blood transfusion ^27^. Vaporisation techniques—in particular, the greenlight laser—have evolved over time by increasing the power delivered by the laser to allow faster treatment ^28^. However, the limitations of the vaporisation technique include the lack of tissue retrieved for analysis to ensure that no cancer is present and a higher rate of needing to convert the technique to an alternative in men with large prostate glands. Holmium laser enucleation of the prostate (HOLEP) remains the most rigorously researched technique suitable to all situations and to men with varying degrees of enlargement. Further randomised controlled trials comparing HOLEP with traditional surgery have been performed with clearly decreased morbidity and similar outcomes at one-year follow-up ^29^. The issue of longevity of the HOLEP technique has also been put to rest with articles publishing long-term outcomes out to 10 years showing low rates of re-intervention and continued relief of symptoms ^30^. The unique property of enucleation is the ability to treat any size of gland, in particular in extremely large prostates, which have traditionally required an open incision operation. This is a morbid procedure and avoiding this is a true advantage of the HOLEP procedure ^31^. With the success of HOLEP and in particular the enucleation technique, a number of new lasers and energy sources based on mimicking this technique have been brought to market. The thulium laser has been the most popular of these along with the diode lasers. Limited data are currently available and it remains to be seen whether these lasers will provide an improvement on the established HOLEP technique ^32^.

So which laser technique is best? There are few trials that compare lasers against each other, as up until this point they have been competing against traditional techniques. A recent trial comparing photoselective vaporisation of the prostate and HOLEP in men with small prostate glands showed similar efficacy and is an example of what is required ^33^. In 2015, we do not have the answer as to which is best. This is due to the variation in surgeons’ training, the equipment available, and the individual patient characteristics such as size of prostate and severity of symptoms. Regardless of which is best, it is clear that lasers are here to stay in the management of BPH.

Robotic surgery in urology is well established for treatment of other conditions unrelated to BPH. A recent trend shows increased use of robotic techniques for treatment of large prostates. These techniques are currently restricted to high-volume robotic centres, but a recent published meta-analysis has shown comparability to open surgery with less morbidity ^34^. However, HOLEP has already established itself as the minimally invasive technique of choice for men with large prostates and therefore any future comparative study would need to compare the robotic approach with this modality. Robotics already has a major hurdle when compared with HOLEP, as a recent cost analysis shows that this procedure is significantly more expensive ^35^.

So what does the future hold for the management of BPH? From a pharmacological point of view, it is using the evidence that male urinary symptoms are not based solely on prostate obstruction. Complex interactions between multiple factors, including bladder receptors, neural pathways, and structural changes, are the targets for future medications. Using combinations of drugs, including anticholinergics and agents such as mirabegron, and the synergies between them will bring increased benefit to men’s urinary symptoms, particularly those that are refractory to standard medication. Novel surgical techniques remain experimental and chase the goal of optimal patient outcomes. Currently, also in clinical trials, water jet ablation is an exciting prospect for the future ^36^. Using the efficient properties or water for tissue ablation and the precision of robotic technology, this procedure is termed aquablation. A large randomised study is under way.

The management of BPH in 2015 is an exciting area to be involved in. Despite all the new medical and surgical options available to the treating doctor, the key message remains that there is not a one-size-fits-all approach. An elderly man with a very large prostate who ends up in urinary retention with a catheter will have different requirements than a young sexually active man with worsening of his symptoms over time causing significant social impact. Both are affected by clinical BPH, but it is up to the physician, in discussion with the patient, to come up with an individual treatment plan suited to their needs. With new technology, this will allow us to do this more efficiently with fewer side effects.
